# Crystal structure and Hirshfeld surface analysis of bis­(2-amino-1-methyl­benzimidazole-κ*N*^3^)bis­(salicylato-κ^2^*O*,*O*′)copper(II)

**DOI:** 10.1107/S2056989025007960

**Published:** 2025-09-19

**Authors:** Salmon Mukhammadiev, Feruza Fayzullayeva, Zukhra Kadirova, Akmaljon Tojiboev, Jamshid Ashurov, Shahlo Daminova

**Affiliations:** a Uzbekistan Japan Innovation Center of Youth, University Street 2B, Tashkent 100095, Uzbekistan; b University of Geological Sciences, Olimlar street, 64, Mirzo Ulug‘bek district, Tashkent, Uzbekistan; c Institute of Bioorganic Chemistry, Academy of Sciences of Uzbekistan, 100125, M.,Ulug‘bek Str, 83, Tashkent, Uzbekistan; d Uzbekistan Japan Innovation Center of Youth University Street 2B Tashkent 100095 National University of Uzbekistan named after Mirzo Ulugbek University Street 4 Tashkent 100174 Uzbekistan; Vienna University of Technology, Austria

**Keywords:** crystal structure, copper(II) complex, 2-amino-1-methyl­benzimidazole, salicylate ligand, void analysis, supra­molecular inter­actions, hydrogen bonding

## Abstract

The title complex, [Cu(C_7_H_5_O_3_)_2_(C_8_H_9_N_3_)_2_], crystallizes in the monoclinic space group *C*2/*c*. The Cu^II^ cation is located on an inversion center and adopts a distorted octa­hedral coordination environment defined by two aromatic N atoms from neutral 2-amino-1-methyl­benzimidazole ligands and by four O atoms from two bidentate salicylate anions coordinating *via* their carboxyl­ate groups. The tri-periodic supra­molecular network features intra- and inter­molecular O—H⋯O, N—H⋯O and C—H⋯O hydrogen bonds, along with π–π stacking and C—H⋯π inter­actions. Hirshfeld surface analysis indicates that H⋯H (43.7% contribution), C⋯H/H⋯C (35.8%), and O⋯H/H⋯O (14.1%) contacts dominate the inter­molecular inter­actions.

## Abstract 2

In the title compound, the Cu^II^ cation lies on an inversion center and exhibits a distorted octa­hedral coordination geometry, formed by two 2-amino-1-methyl­benzimidazole ligands coordinating *via* their ring nitro­gen atom and two bidentate salicylate anions binding through the carboxyl­ate oxygen atoms.

## 1. Chemical context

Salicylic acid or derivatives thereof form stable complexes with the central metal cation (*e.g.* copper) *via* strong coordination bonds either in a monodentate or bidentate fashion, forming bonds to the oxygen atoms of the carboxyl group and/or the hydroxyl group (Iravani *et al.*, 2013 ▸; Hoang *et al.*, 1992 ▸). Corresponding complexes exhibit distinctive magnetic and optical properties, influenced by the electronic configuration of the central metal cation and the π-electron system of the ligand, thereby enhancing potential applications in catalysis, optical materials, or biological systems (Costes *et al.*, 2003 ▸).

Copper(II) complexes formed by salicylic acid and a co-ligand, such as benzimidazole and its derivatives, have continued to attract attention due to their structural diversity and biological potential. In these mixed N,O-donor ligand systems, salicylic acid can coordinate via its hydroxyl and carboxyl­ate groups, while aromatic amines bind through nitro­gen atoms (Lawal *et al.*, 2017 ▸). These combinations of ligands provide favorable coordination environments, influencing the crystal packing and stability of the resulting complexes.

**Figure 1:**
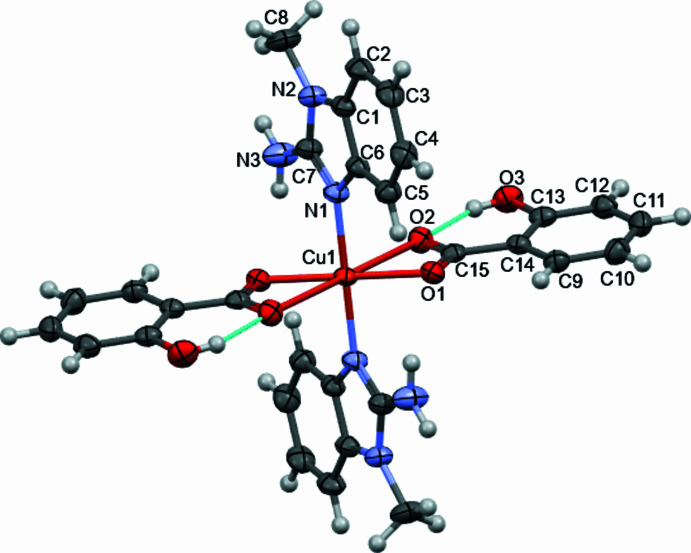
Mol­ecular structure of the title complex. Displacement ellipsoids are drawn at the 50% probability level; non-labeled atoms are generated by symmetry operation 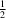 − *x*, 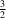 − *y*, 1 − *z*.

In the context given above, we report here on the crystal structure, Hirshfeld and void analysis of a new Cu^II^ complex derived from salicylic acid and benzimidazole, [Cu(C_7_H_5_O_3_)_2_(C_8_H_9_N_3_)_2_] (Fig. 1 ▸).
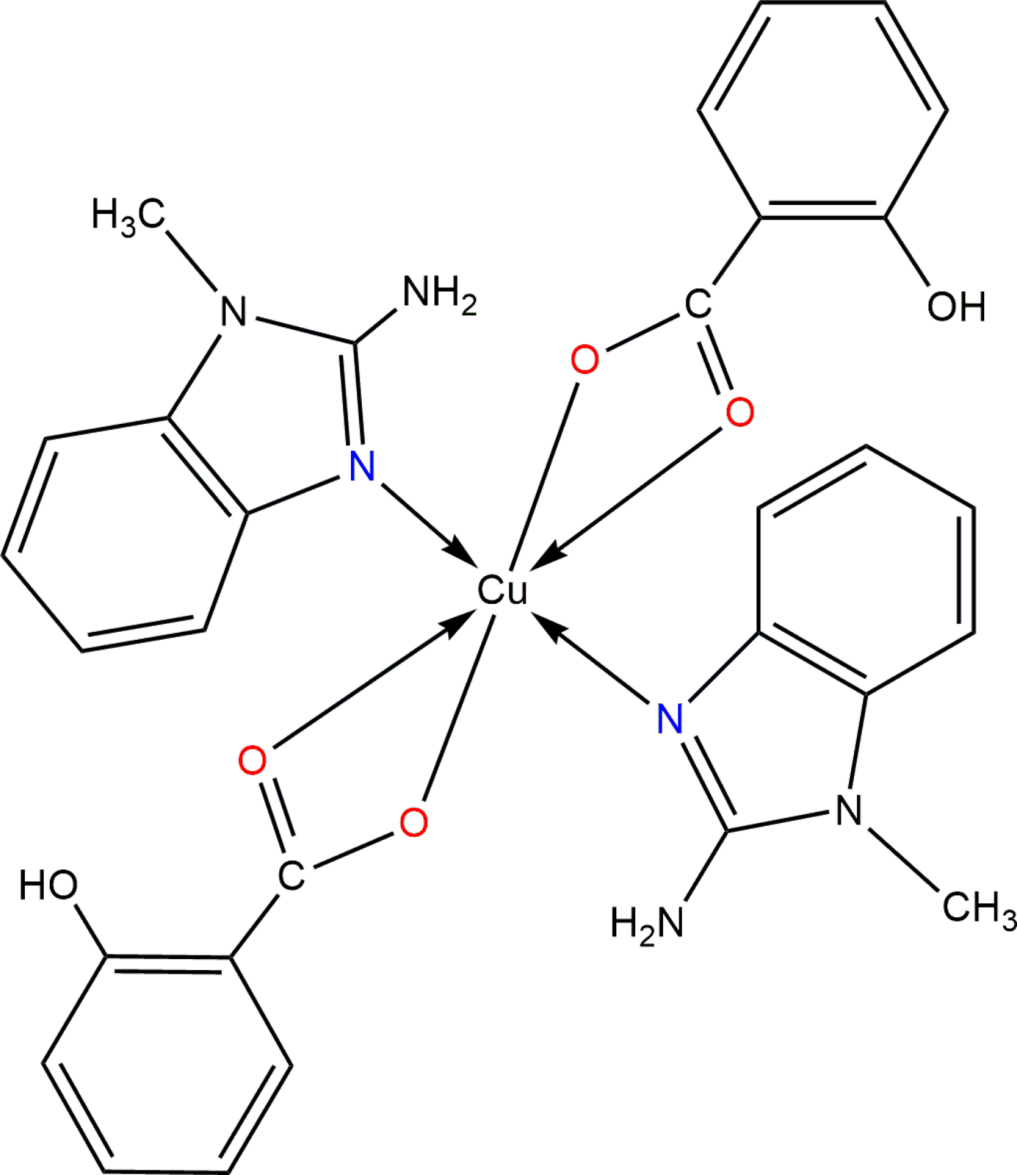


## 2. Structural commentary

The asymmetric unit of the title complex comprises one half of the [Cu(C_7_H_5_O_3_)_2_(C_8_H_9_N_3_)_2_] mol­ecule, the complete complex being generated by inversion symmetry. The central Cu^II^ cation adopts a distorted octa­hedral coordination environment defined by two nitro­gen atoms [N1, N1^i^; symmetry code: (i) −*x* + 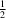, −*y* + 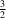, −*z* + 1] from two neutral 2-amino-1-methyl­benzimidazole (MAB) ligands and four oxygen atoms from two bidentate salicylate ligands, coordinating in a *κ*^2^O,O′ fashion (O1, O2; O1^i^, O2^i^) through the carboxyl­ate groups. The Cu—N and Cu—O bond lengths fall within expected ranges for the typical Jahn–Teller distortion, with the Cu1—N1, Cu1—O1, and Cu1—O2 distances being 1.9781 (16), 1.9849 (14), and 2.5672 (15) Å, respectively. The salicylate ligand forms a four-membered chelate ring with a bite angle of 56.60 (5)° for O1—Cu1—O2 and a *cis* angle of 123.40 (5)° for O1—Cu1—O2^i^. Within the salicylate ligand an intra­molecular O—H⋯O hydrogen bond between the phenolic hydroxyl group (O3—H3) and the adjacent carboxyl­ate oxygen atom (O2) helps to consolidate the mol­ecular conformation (Table 1 ▸). The N—Cu1—O angles are 88.95 (6) and 91.05 (6)°. These values are consistent with those observed in previously reported Cu^II^ complexes bearing mixed N,O-donor ligands (Puchoňová *et al.*, 2017 ▸). The dihedral angle between the benzimidazole ring of the MAB ligand and the aromatic ring of the coordinated salicylate ligand is 82.68 (11)°.

## 3. Supra­molecular features

**Figure 2:**
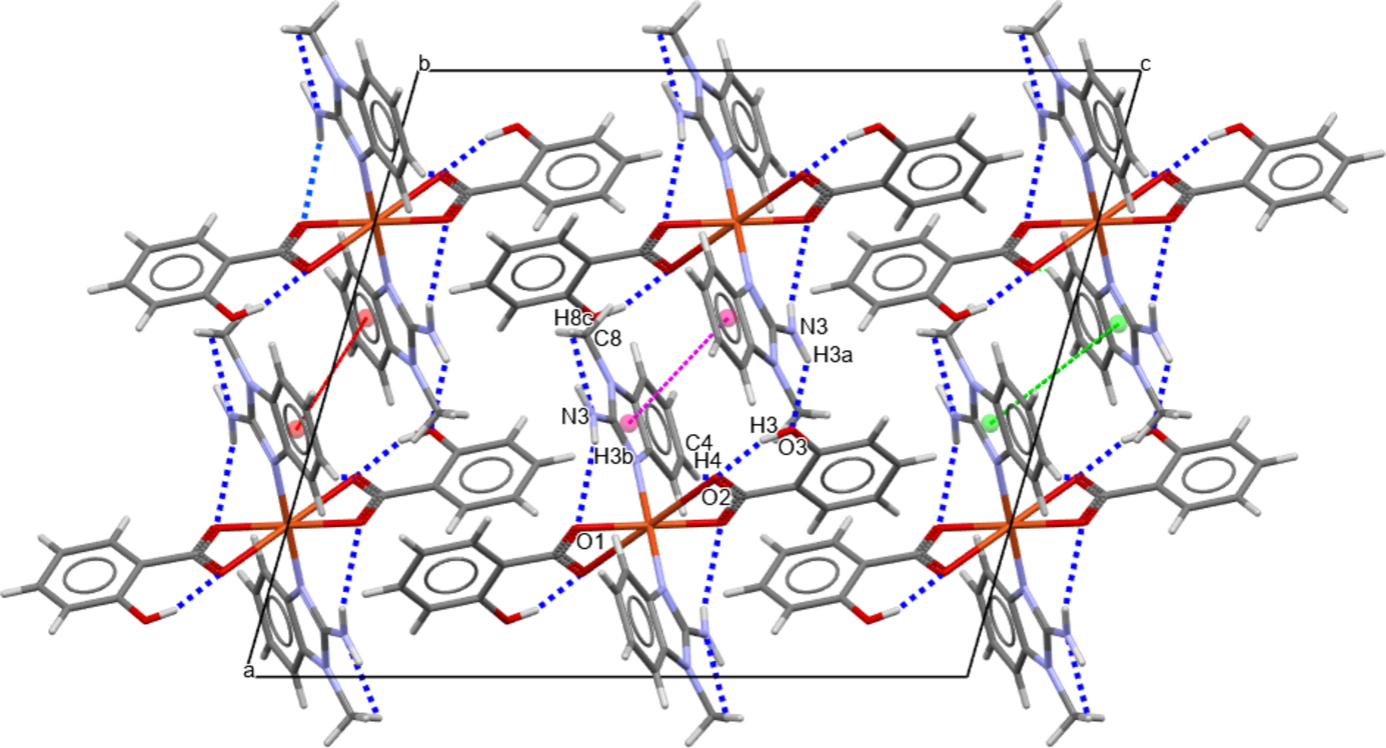
The mol­ecular packing of the title compound along [010]. Intra- and inter­molecular hydrogen bonds are shown as dashed lines. π–π stacking inter­actions are indicated as follows: *Cg*3⋯*Cg*3 in green, *Cg*3⋯*Cg*4 in pink, and *Cg*4⋯*Cg*4 in red.

The title compound exhibits a tri-periodic supra­molecular network defined by a variety of non-covalent inter­actions. Two key inter­molecular N—H⋯O hydrogen bonds (Table 1 ▸) are observed between the amino group of the benzimidazole ligand (N3) and neighboring salicylate oxygen atoms (O3). Additional weaker C—H⋯O contacts further contribute to the supra­molecular cohesion (Table 1 ▸). π–π stacking inter­actions are observed between aromatic rings N1/C6/C1/N2/C7 (centroid *Cg*3) and C1–C6 (*Cg*4) with a centroid-to-centroid distances of 3.8831 (13) Å for *Cg*3⋯*Cg*4(1 − *x*, 1 − *y*, 1 − *z*; slippage = 1.769 Å), as shown in Fig. 2 ▸. Furthermore, a weak C—H⋯π inter­actions is present between the C11—H11 group and the π-systems represented by *Cg*4. The H11⋯*Cg* distance is 2.637 (3), the C11⋯*Cg* distance is 3.543 (3) and the C11—H11⋯*Cg* angle is 159.6 (3)°.

## 4. Hirshfeld surface and void analysis

**Figure 3:**
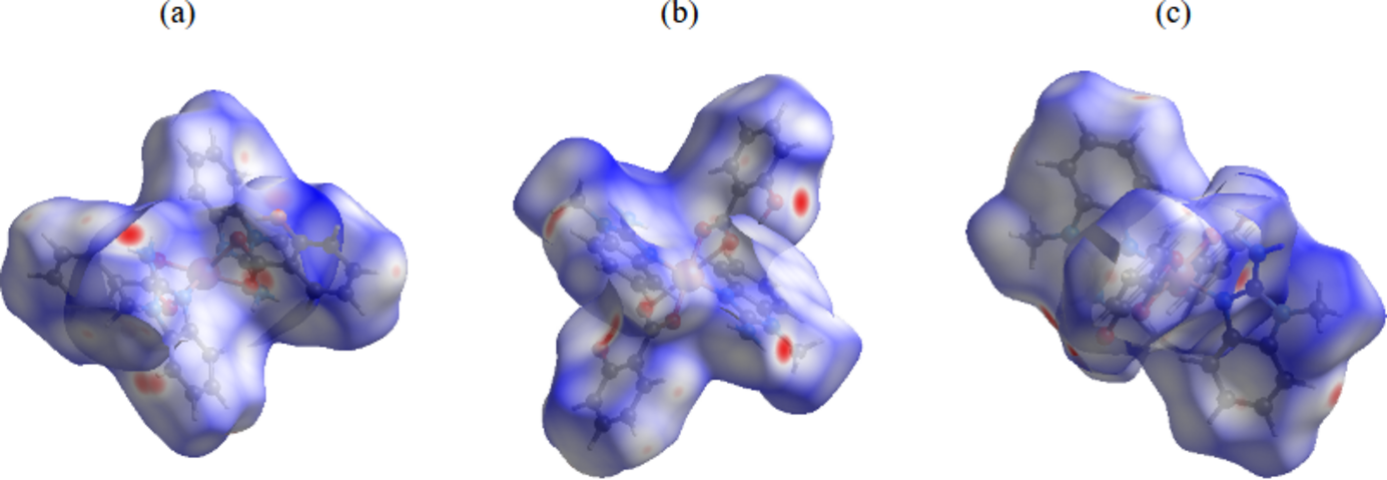
HS plotted over *d*_norm_ (*a*) along [100], (*b*) along [010] and (*c*) along [001].

**Figure 4:**
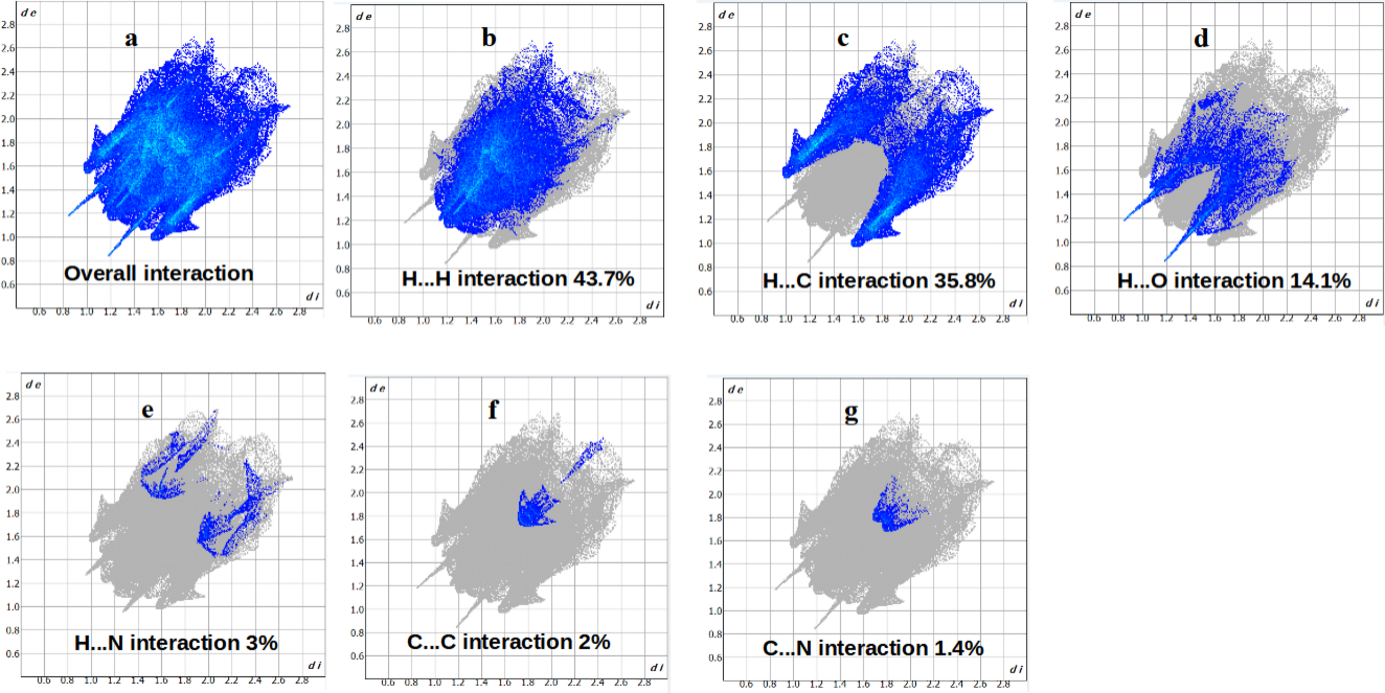
Two-dimensional fingerprint plots for (*a*) all inter­actions and (*b*)–(*g*) individual inter­atomic contacts.

In order to qu­antify and visualize inter­molecular inter­actions, a Hirshfeld surface (HS) analysis (Spackman & Jayatilaka, 2009 ▸) was performed and the associated two-dimensional fingerprint plots (McKinnon *et al.*, 2007 ▸) calculated with *CrystalExplorer*21 (Spackman *et al.*, 2021 ▸). The HS mapped with *d*_norm_ is represented in Fig. 3 ▸, where white regions indicate contacts at van der Waals separations, red spots denote shorter contacts (*e.g.* hydrogen bonds) and blue areas longer contacts. The overall two-dimensional fingerprint plot is shown in Fig. 4 ▸*a*. H⋯H contacts make the largest contribution (43.7%, Fig. 4 ▸*b*) to the HS. Other significant contacts are H⋯C/C⋯H (35.8%, Fig. 4 ▸*c*) and H⋯O/O⋯H (14.1%, Fig. 4 ▸*d*), while H⋯N/N⋯H (3%, Fig. 4 ▸*e*), C⋯C (2%, Fig. 4 ▸*f*) and C⋯N/N⋯C (1.4%, Fig. 4 ▸*g*) contacts contribute only to a minor amount.

**Figure 5:**
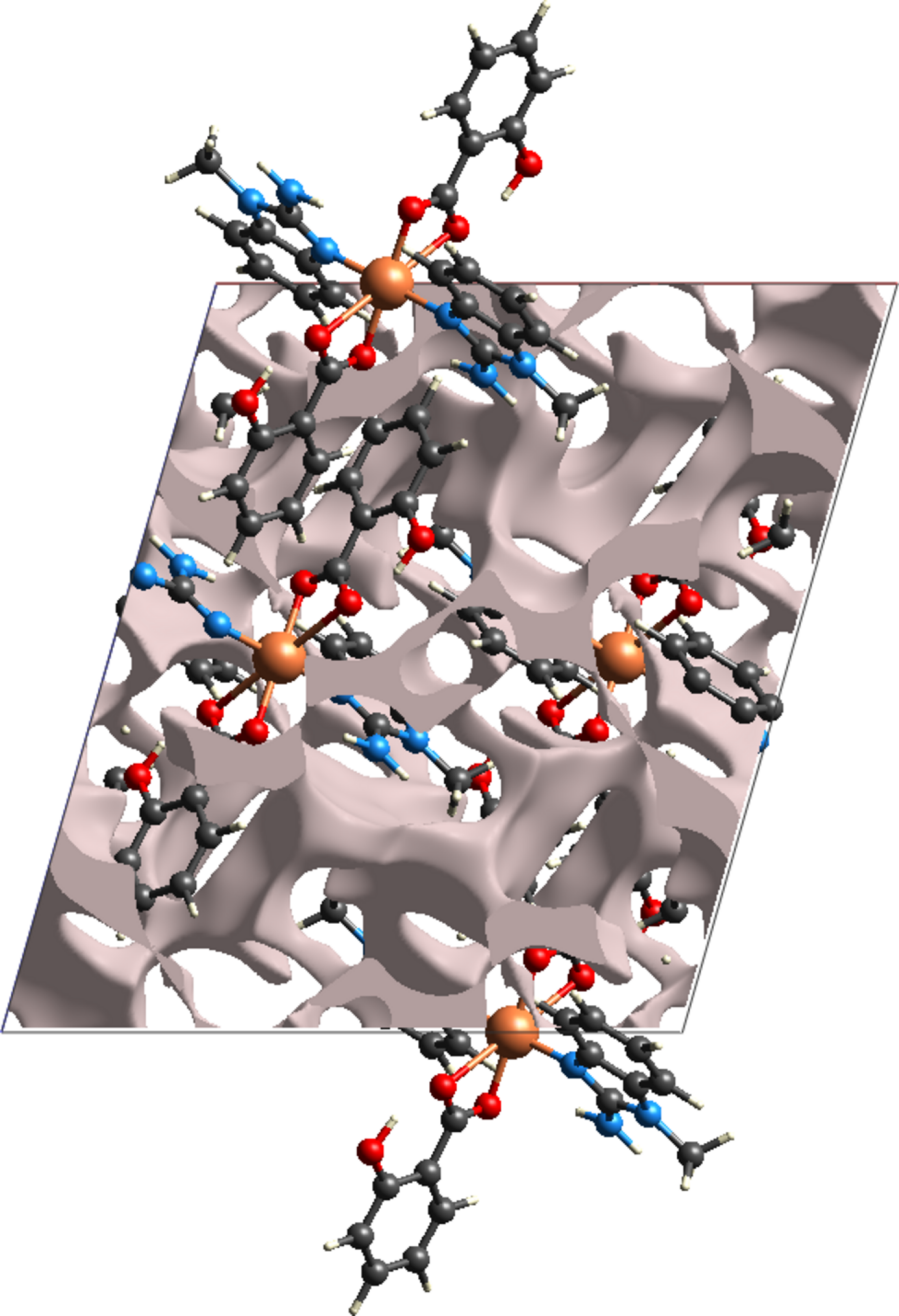
The void surface packing in a view along [010].

Void analysis was performed using *CrystalExplorer* with a probe radius of 1.2 Å and a grid spacing of 0.2 Å. The total void volume within the unit cell was calculated to be 327.6 Å^3^, which corresponds to 11.7% of the unit-cell volume. The voids are visualized as transparent isosurfaces in the crystal packing diagram (Fig. 5 ▸).

## 5. Database survey

A search of the Cambridge Structural Database (CSD, version 5.46, updated November 2024; Groom *et al.*, 2016 ▸) for similar coordination environments (bidentate carboxyl­ate group and an aromatic monodentate N-donor ligand) yielded several complexes including the first-row transition metal complex: *catena*-[bis­(μ-carbonato)tetra­kis­(2,4′-bi­pyridine)­bis­(aqua)­dicopper(II) dihydrate] (RITBEE01; Mulrooney *et al.*, 2018 ▸), which features a bidentate binding and bridging carbonate anion and monodentately binding 2,2′-bi­pyridine entities, resulting in the formation of a polymeric chain.

## 6. Synthesis and crystallization

An aqueous solution of copper(II) sulfate penta­hydrate (0.05 *M*, 10 ml) was prepared by dissolving the salt in distilled water. An ethano­lic solution of salicylic acid (0.1 *M*, 10 ml) was added, and the resulting mixture stirred magnetically at room temperature for 4 h. No noticeable color change was observed during this step. Subsequently, an ethano­lic solution of 2-amino-1-methyl­benzimidazole (0.1 *M*, 10 ml) was added, and stirring was continued for additional 4 h, resulting in a green-colored solution. The reaction mixture was then filtered and left to stand at room temperature for 2–3 weeks to allow the growth of green crystals suitable for X-ray diffraction analysis.

## 7. Refinement

Crystal data, data collection and structure refinement details are summarized in Table 2 ▸. Hydrogen atoms were positioned geometrically (aromatic C—H = 0.95 Å, N—H = 0.89 Å, O—H = 0.84 Å and methyl C—H = 0.98 Å) and refined using a riding model with *U*_iso_(H) = 1.2*U*_eq_(aromatic C, N) or 1.5*U*_eq_(methyl C, O).

## Supplementary Material

Crystal structure: contains datablock(s) I. DOI: 10.1107/S2056989025007960/wm5761sup1.cif

CCDC reference: 2485535

Additional supporting information:  crystallographic information; 3D view; checkCIF report

## Figures and Tables

**Table 1:** Hydrogen-bond geometry (Å, °)

*D*—H⋯*A*	*D*—H	H⋯*A*	*D*⋯*A*	*D*—H⋯*A*
O3—H3⋯O2	0.81 (1)	1.81 (1)	2.538 (2)	150 (1)
N3—H3*a*⋯O3^i^	0.88 (1)	2.33 (1)	3.046 (3)	139 (1)
N3—H3*b*⋯O1	0.88 (1)	2.49 (1)	3.020 (3)	119 (1)
C4—H4⋯O2^ii^	0.95 (1)	2.57 (1)	3.373 (2)	143 (1)

Symmetry codes: (i) 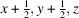; (ii) 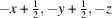.

**Table 2:** Experimental details

Crystal data	
Chemical formula	[Cu(C_7_H_5_O_3_)_2_(C_8_H_9_N_3_)_2_]
*M*_r_	632.14
Crystal system, space group	Monoclinic, *C*2/*c*
Temperature (K)	150
*a*, *b*, *c* (Å)	16.7892 (5), 9.0355 (2), 19.2315 (6)
β (°)	106.011 (3)
*V* (Å^3^)	2804.23 (14)
*Z*	4
Radiation type	Cu *K*α
μ (mm^−1^)	1.58
Crystal size (mm)	0.54 × 0.18 × 0.06

Data collection	
Diffractometer	XtaLAB Synergy, Single source at home/near, HyPix3000
Absorption correction	Multi-scan (*CrysAlis PRO*; Rigaku OD, 2023 ▸)
*T*_min_, *T*_max_	0.710, 1.000
No. of measured, independent and observed [*I* ≥ 2u(*I*)] reflections	13358, 2710, 2351
*R*_int_	0.046
(sin θ/λ)_max_ (Å^−1^)	0.616

Refinement	
*R*[*F*^2^ > 2σ(*F*^2^)], *wR*(*F*^2^), *S*	0.038, 0.108, 1.02
No. of reflections	2710
No. of parameters	197
H-atom treatment	H-atom parameters constrained
Δρ_max_, Δρ_min_ (e Å^−3^)	0.36, −0.44

Computer programs: *CrysAlis PRO* (Rigaku OD, 2023 ▸), *OLEX2.solve* (Bourhis *et al.*, 2015 ▸), *SHELXL* (Sheldrick, 2015 ▸) and *OLEX2* (Dolomanov *et al.*, 2009 ▸).

## supplementary crystallographic information

### Bis(2-hydroxybenzoato-κ2O1,O1')bis(1-methyl-1H-1,3-benzodiazol-2-amine-κN3)copper(II) Crystal data

**Table:**

[Cu(C_7_H_5_O_3_)_2_(C_8_H_9_N_3_)_2_]	*F*(000) = 1308.0
*M**_r_* = 632.14	*D*_x_ = 1.497 Mg m^−^^3^
Monoclinic, *C*2/*c*	Cu *K*α radiation, λ = 1.54184 Å
*a* = 16.7892 (5) Å	Cell parameters from 6926 reflections
*b* = 9.0355 (2) Å	θ = 4.8–71.0°
*c* = 19.2315 (6) Å	µ = 1.58 mm^−^^1^
β = 106.011 (3)°	*T* = 150 K
*V* = 2804.23 (14) Å^3^	Block, dark green
*Z* = 4	0.54 × 0.18 × 0.06 mm

### Bis(2-hydroxybenzoato-κ2O1,O1')bis(1-methyl-1H-1,3-benzodiazol-2-amine-κN3)copper(II) Data collection

**Table:**

XtaLAB Synergy, Single source at home/near, HyPix3000 diffractometer	2710 independent reflections
Radiation source: micro-focus sealed X-ray tube, PhotonJet (Cu) X-ray Source	2351 reflections with *I*≥ 2u(*I*)
Mirror monochromator	*R*_int_ = 0.046
Detector resolution: 10.0000 pixels mm^-1^	θ_max_ = 71.7°, θ_min_ = 4.8°
ω scans	*h* = −20→17
Absorption correction: multi-scan (CrysAlisPro; Rigaku OD, 2023)	*k* = −11→10
*T*_min_ = 0.710, *T*_max_ = 1.000	*l* = −23→23
13358 measured reflections	

### Bis(2-hydroxybenzoato-κ2O1,O1')bis(1-methyl-1H-1,3-benzodiazol-2-amine-κN3)copper(II) Refinement

**Table:**

Refinement on *F*^2^	0 restraints
Least-squares matrix: full	25 constraints
*R*[*F*^2^ > 2σ(*F*^2^)] = 0.038	H-atom parameters constrained
*wR*(*F*^2^) = 0.108	*w* = 1/[σ^2^(*F*_o_^2^) + (0.0592*P*)^2^ + 2.8103*P*] where *P* = (*F*_o_^2^ + 2*F*_c_^2^)/3
*S* = 1.02	(Δ/σ)_max_ = −0.001
2710 reflections	Δρ_max_ = 0.36 e Å^−^^3^
197 parameters	Δρ_min_ = −0.44 e Å^−^^3^

### Bis(2-hydroxybenzoato-κ2O1,O1')bis(1-methyl-1H-1,3-benzodiazol-2-amine-κN3)copper(II) Fractional atomic coordinates and isotropic or equivalent isotropic displacement parameters (Å^2^)

**Table:**

	*x*	*y*	*z*	*U*_iso_*/*U*_eq_	
Cu1	0.25	0.75	0.5	0.02441 (15)	
O1	0.24551 (8)	0.80636 (16)	0.59860 (8)	0.0287 (3)	
O2	0.17132 (9)	0.60031 (16)	0.57333 (8)	0.0331 (4)	
O3	0.09241 (9)	0.50418 (17)	0.65918 (9)	0.0385 (4)	
H3	0.10951 (9)	0.50901 (17)	0.62385 (9)	0.0577 (6)*	
N1	0.35479 (10)	0.64351 (19)	0.54380 (9)	0.0284 (4)	
N2	0.48415 (10)	0.5937 (2)	0.60893 (10)	0.0321 (4)	
N3	0.43028 (12)	0.8317 (2)	0.62124 (12)	0.0469 (6)	
H3a	0.47667 (12)	0.8575 (2)	0.65315 (12)	0.0563 (7)*	
H3b	0.38887 (12)	0.8948 (2)	0.60864 (12)	0.0563 (7)*	
C1	0.45469 (12)	0.4673 (2)	0.57009 (12)	0.0292 (5)	
C2	0.49118 (14)	0.3304 (3)	0.56638 (13)	0.0367 (5)	
H2	0.54675 (14)	0.3105 (3)	0.59311 (13)	0.0441 (6)*	
C3	0.44333 (16)	0.2248 (3)	0.52229 (14)	0.0401 (6)	
H3c	0.46642 (16)	0.1301 (3)	0.51867 (14)	0.0481 (7)*	
C4	0.36121 (16)	0.2540 (2)	0.48257 (14)	0.0386 (5)	
H4	0.32941 (16)	0.1784 (2)	0.45334 (14)	0.0463 (7)*	
C5	0.32582 (14)	0.3926 (2)	0.48553 (12)	0.0334 (5)	
H5	0.27066 (14)	0.4134 (2)	0.45803 (12)	0.0401 (6)*	
C6	0.37341 (12)	0.4982 (2)	0.52959 (11)	0.0276 (4)	
C7	0.42262 (12)	0.6952 (2)	0.59162 (12)	0.0308 (5)	
C8	0.56419 (14)	0.6094 (3)	0.66364 (14)	0.0458 (6)	
H8a	0.5682 (5)	0.5360 (14)	0.7020 (5)	0.0686 (9)*	
H8b	0.60917 (14)	0.594 (2)	0.6410 (3)	0.0686 (9)*	
H8c	0.5687 (5)	0.7091 (8)	0.6844 (8)	0.0686 (9)*	
C9	0.22167 (13)	0.8228 (2)	0.73737 (12)	0.0310 (5)	
H9	0.25920 (13)	0.8908 (2)	0.72568 (12)	0.0373 (5)*	
C10	0.20417 (14)	0.8346 (3)	0.80299 (12)	0.0380 (5)	
H10	0.22939 (14)	0.9099 (3)	0.83627 (12)	0.0456 (6)*	
C11	0.14918 (15)	0.7351 (3)	0.82013 (13)	0.0387 (6)	
H11	0.13671 (15)	0.7428 (3)	0.86527 (13)	0.0464 (7)*	
C12	0.11264 (13)	0.6251 (3)	0.77193 (12)	0.0362 (5)	
H12	0.07531 (13)	0.5577 (3)	0.78424 (12)	0.0434 (6)*	
C13	0.13000 (12)	0.6125 (2)	0.70566 (12)	0.0298 (5)	
C14	0.18537 (12)	0.7131 (2)	0.68769 (11)	0.0259 (4)	
C15	0.20180 (12)	0.7050 (2)	0.61594 (11)	0.0270 (4)	

### Bis(2-hydroxybenzoato-κ2O1,O1')bis(1-methyl-1H-1,3-benzodiazol-2-amine-κN3)copper(II) Atomic displacement parameters (Å^2^)

**Table:**

	*U*^11^	*U*^22^	*U*^33^	*U*^12^	*U*^13^	*U*^23^
Cu1	0.0213 (2)	0.0226 (2)	0.0293 (2)	0.00500 (15)	0.00676 (17)	0.00076 (16)
O1	0.0257 (7)	0.0291 (7)	0.0324 (8)	0.0017 (6)	0.0101 (6)	0.0006 (6)
O2	0.0384 (8)	0.0271 (8)	0.0333 (8)	0.0027 (6)	0.0088 (6)	−0.0039 (6)
O3	0.0366 (8)	0.0323 (8)	0.0444 (9)	−0.0093 (7)	0.0078 (7)	−0.0011 (7)
N1	0.0253 (8)	0.0253 (9)	0.0344 (9)	0.0051 (7)	0.0077 (7)	−0.0008 (7)
N2	0.0231 (8)	0.0331 (10)	0.0371 (10)	0.0025 (7)	0.0033 (7)	0.0011 (8)
N3	0.0320 (10)	0.0344 (11)	0.0650 (14)	0.0041 (8)	−0.0020 (9)	−0.0167 (10)
C1	0.0251 (10)	0.0291 (11)	0.0348 (11)	0.0050 (8)	0.0108 (8)	0.0060 (9)
C2	0.0328 (11)	0.0354 (12)	0.0432 (13)	0.0124 (9)	0.0124 (10)	0.0083 (10)
C3	0.0488 (14)	0.0290 (12)	0.0448 (13)	0.0147 (10)	0.0169 (11)	0.0021 (10)
C4	0.0480 (14)	0.0267 (12)	0.0409 (13)	0.0040 (9)	0.0121 (11)	−0.0047 (9)
C5	0.0329 (11)	0.0305 (11)	0.0355 (12)	0.0043 (9)	0.0073 (9)	−0.0015 (9)
C6	0.0281 (10)	0.0248 (10)	0.0314 (11)	0.0054 (8)	0.0107 (8)	0.0030 (8)
C7	0.0250 (10)	0.0296 (11)	0.0373 (12)	0.0029 (8)	0.0075 (8)	−0.0013 (9)
C8	0.0262 (11)	0.0501 (15)	0.0516 (15)	0.0027 (10)	−0.0052 (10)	0.0012 (12)
C9	0.0267 (10)	0.0312 (11)	0.0339 (11)	−0.0019 (8)	0.0062 (8)	0.0003 (9)
C10	0.0397 (12)	0.0388 (13)	0.0322 (12)	0.0003 (10)	0.0042 (10)	−0.0056 (10)
C11	0.0381 (12)	0.0495 (15)	0.0300 (12)	0.0081 (10)	0.0119 (10)	0.0049 (10)
C12	0.0290 (10)	0.0412 (13)	0.0394 (12)	0.0017 (9)	0.0111 (9)	0.0119 (10)
C13	0.0242 (9)	0.0261 (10)	0.0364 (11)	0.0023 (8)	0.0038 (8)	0.0041 (9)
C14	0.0212 (9)	0.0251 (10)	0.0309 (11)	0.0030 (7)	0.0063 (8)	0.0022 (8)
C15	0.0223 (9)	0.0251 (10)	0.0315 (11)	0.0074 (8)	0.0039 (8)	0.0026 (8)

### Bis(2-hydroxybenzoato-κ2O1,O1')bis(1-methyl-1H-1,3-benzodiazol-2-amine-κN3)copper(II) Geometric parameters (Å, º)

**Table:**

Cu1—O1	1.9849 (14)	C3—H3c	0.9500
Cu1—O1^i^	1.9849 (14)	C3—C4	1.405 (3)
Cu1—O2	2.5672 (15)	C4—H4	0.9500
Cu1—N1	1.9781 (16)	C4—C5	1.394 (3)
Cu1—N1^i^	1.9781 (16)	C5—H5	0.9500
O1—C15	1.274 (3)	C5—C6	1.376 (3)
O2—C15	1.264 (3)	C8—H8a	0.9800
O3—H3	0.8084	C8—H8b	0.9800
O3—C13	1.358 (3)	C8—H8c	0.9800
N1—C6	1.394 (3)	C9—H9	0.9500
N1—C7	1.335 (3)	C9—C10	1.376 (3)
N2—C1	1.380 (3)	C9—C14	1.395 (3)
N2—C7	1.352 (3)	C10—H10	0.9500
N2—C8	1.467 (3)	C10—C11	1.392 (3)
N3—H3a	0.8800	C11—H11	0.9500
N3—H3b	0.8800	C11—C12	1.381 (3)
N3—C7	1.350 (3)	C12—H12	0.9500
C1—C2	1.391 (3)	C12—C13	1.388 (3)
C1—C6	1.400 (3)	C13—C14	1.410 (3)
C2—H2	0.9500	C14—C15	1.482 (3)
C2—C3	1.377 (4)		
			
O1—Cu1—O2^i^	123.40 (5)	C1—C6—N1	108.42 (18)
O1—Cu1—O2	56.60 (5)	C5—C6—N1	130.57 (19)
O1^i^—Cu1—O1	180.0	C5—C6—C1	121.01 (19)
N1^i^—Cu1—O1^i^	88.95 (6)	N2—C7—N1	112.40 (19)
N1^i^—Cu1—O1	91.05 (6)	N3—C7—N1	125.03 (19)
N1—Cu1—O1^i^	91.05 (6)	N3—C7—N2	122.56 (19)
N1—Cu1—O1	88.95 (6)	H8a—C8—N2	109.5
N1^i^—Cu1—N1	180.0	H8b—C8—N2	109.5
C15—O1—Cu1	104.01 (13)	H8b—C8—H8a	109.5
C13—O3—H3	107.8	H8c—C8—N2	109.5
C6—N1—Cu1	126.57 (14)	H8c—C8—H8a	109.5
C7—N1—Cu1	127.78 (14)	H8c—C8—H8b	109.5
C7—N1—C6	105.64 (16)	C10—C9—H9	119.36 (13)
C7—N2—C1	107.01 (17)	C14—C9—H9	119.36 (13)
C8—N2—C1	126.06 (19)	C14—C9—C10	121.3 (2)
C8—N2—C7	126.7 (2)	H10—C10—C9	120.31 (13)
H3b—N3—H3a	120.0	C11—C10—C9	119.4 (2)
C7—N3—H3a	120.0	C11—C10—H10	120.31 (14)
C7—N3—H3b	120.0	H11—C11—C10	119.79 (14)
C2—C1—N2	131.8 (2)	C12—C11—C10	120.4 (2)
C6—C1—N2	106.52 (18)	C12—C11—H11	119.79 (14)
C6—C1—C2	121.6 (2)	H12—C12—C11	119.72 (14)
H2—C2—C1	121.41 (13)	C13—C12—C11	120.6 (2)
C3—C2—C1	117.2 (2)	C13—C12—H12	119.72 (13)
C3—C2—H2	121.41 (13)	C12—C13—O3	119.12 (19)
H3c—C3—C2	119.23 (13)	C14—C13—O3	121.4 (2)
C4—C3—C2	121.5 (2)	C14—C13—C12	119.4 (2)
C4—C3—H3c	119.23 (13)	C13—C14—C9	118.9 (2)
H4—C4—C3	119.61 (13)	C15—C14—C9	120.95 (19)
C5—C4—C3	120.8 (2)	C15—C14—C13	120.10 (19)
C5—C4—H4	119.61 (14)	O2—C15—O1	121.9 (2)
H5—C5—C4	121.09 (14)	C14—C15—O1	118.49 (18)
C6—C5—C4	117.8 (2)	C14—C15—O2	119.64 (19)
C6—C5—H5	121.09 (12)		
			
Cu1—O1—C15—O2	−0.95 (13)	N3—C7—N2—C1	178.8 (2)
Cu1—O1—C15—C14	−179.75 (10)	N3—C7—N2—C8	4.1 (3)
Cu1—N1—C6—C1	178.22 (18)	C1—C2—C3—C4	0.1 (3)
Cu1—N1—C6—C5	−2.4 (2)	C1—C6—N1—C7	−0.6 (2)
Cu1—N1—C7—N2	−178.35 (19)	C1—C6—C5—C4	0.0 (2)
Cu1—N1—C7—N3	2.8 (2)	C2—C1—N2—C7	179.4 (3)
O1—C15—C14—C9	−4.6 (2)	C2—C1—N2—C8	−5.8 (3)
O1—C15—C14—C13	173.06 (17)	C2—C1—C6—C5	1.4 (3)
O2—C15—C14—C9	176.60 (18)	C2—C3—C4—C5	1.2 (3)
O2—C15—C14—C13	−5.8 (2)	C3—C2—C1—C6	−1.4 (3)
O3—C13—C12—C11	−179.33 (19)	C3—C4—C5—C6	−1.2 (3)
O3—C13—C14—C9	179.50 (19)	C5—C6—N1—C7	178.7 (3)
O3—C13—C14—C15	1.8 (2)	C6—C1—N2—C7	−0.2 (2)
N1—C6—C1—N2	0.55 (18)	C6—C1—N2—C8	174.49 (18)
N1—C6—C1—C2	−179.18 (18)	C9—C10—C11—C12	0.2 (3)
N1—C6—C5—C4	−179.3 (3)	C9—C14—C13—C12	0.3 (2)
N1—C7—N2—C1	−0.2 (2)	C10—C9—C14—C13	−0.2 (2)
N1—C7—N2—C8	−174.86 (19)	C10—C9—C14—C15	177.42 (19)
N2—C1—C2—C3	178.9 (3)	C10—C11—C12—C13	−0.2 (3)
N2—C1—C6—C5	−178.89 (17)	C11—C10—C9—C14	0.0 (3)
N2—C7—N1—C6	0.5 (2)	C11—C12—C13—C14	−0.1 (3)
N3—C7—N1—C6	−178.4 (3)	C12—C13—C14—C15	−177.40 (18)

Symmetry code: (i) −*x*+1/2, −*y*+3/2, −*z*+1.

### Bis(2-hydroxybenzoato-κ2O1,O1')bis(1-methyl-1H-1,3-benzodiazol-2-amine-κN3)copper(II) Hydrogen-bond geometry (Å, º)

**Table:**

*D*—H···*A*	*D*—H	H···*A*	*D*···*A*	*D*—H···*A*
O3—H3···O2	0.81 (1)	1.81 (1)	2.538 (2)	150 (1)
N3—H3*a*···O3^ii^	0.88 (1)	2.33 (1)	3.046 (3)	139 (1)
N3—H3*b*···O1	0.88 (1)	2.49 (1)	3.020 (3)	119 (1)
C4—H4···O2^iii^	0.95 (1)	2.57 (1)	3.373 (2)	143 (1)
C8—H8*c*···N3	0.98 (1)	2.56 (1)	2.957 (3)	104 (1)

Symmetry codes: (ii) *x*+1/2, *y*+1/2, *z*; (iii) −*x*+1/2, −*y*+1/2, −*z*.
